# Towards causal mechanisms of consciousness through focused transcranial brain stimulation

**DOI:** 10.1093/nc/niad008

**Published:** 2023-04-21

**Authors:** Marek Havlík, Jaroslav Hlinka, Monika Klírová, Petr Adámek, Jiří Horáček

**Affiliations:** Center for Advanced Studies of Brain and Consciousness, National Institute of Mental Health, Topolová 748, Klecany 250 67, Czech Republic; Center for Advanced Studies of Brain and Consciousness, National Institute of Mental Health, Topolová 748, Klecany 250 67, Czech Republic; Department of Complex Systems, Institute of Computer Science of the Czech Academy of Sciences, Pod Vodárenskou věží 271/2, Prague 182 07, Czech Republic; Center for Advanced Studies of Brain and Consciousness, National Institute of Mental Health, Topolová 748, Klecany 250 67, Czech Republic; Third Faculty of Medicine, Charles University, Ruská 87, Prague 10 100 00, Czech Republic; Center for Advanced Studies of Brain and Consciousness, National Institute of Mental Health, Topolová 748, Klecany 250 67, Czech Republic; Third Faculty of Medicine, Charles University, Ruská 87, Prague 10 100 00, Czech Republic; Center for Advanced Studies of Brain and Consciousness, National Institute of Mental Health, Topolová 748, Klecany 250 67, Czech Republic; Third Faculty of Medicine, Charles University, Ruská 87, Prague 10 100 00, Czech Republic

**Keywords:** consciousness, neural correlates of consciousness, mechanisms of consciousness, causal explanation, excitation, inhibition, brain stimulation, transcranial electric stimulation, geodesic transcranial electric neuromodulation

## Abstract

Conscious experience represents one of the most elusive problems of empirical science, namely neuroscience. The main objective of empirical studies of consciousness has been to describe the minimal sets of neural events necessary for a specific neuronal state to become consciously experienced. The current state of the art still does not meet this objective but rather consists of highly speculative theories based on correlates of consciousness and an ever-growing list of knowledge gaps. The current state of the art is defined by the limitations of past stimulation techniques and the emphasis on the observational approach. However, looking at the current stimulation technologies that are becoming more accurate, it is time to consider an alternative approach to studying consciousness, which builds on the methodology of causal explanations via causal alterations. The aim of this methodology is to move beyond the correlates of consciousness and focus directly on the mechanisms of consciousness with the help of the currently focused brain stimulation techniques, such as geodesic transcranial electric neuromodulation. This approach not only overcomes the limitations of the correlational methodology but will also become another firm step in the following science of consciousness.

## Introduction

Conscious experience is the ongoing and ever-changing stream of various mental contents, such as sensations, emotions, thoughts, and all the other mental states that become conscious. It is a united representation of both the outside and the inner world. Although consciousness is probably only a small fraction of the entire physiological unconscious machinery of the brain, it is the only thing through which we have access to brain-generated representations of the outside world, our predictions, and interpretations of events. Paradoxically, each human being knows conscious experience most intimately and better than anything else; on the other hand, conscious experience is one of the most elusive problems of current empirical science. The main reason for this elusiveness lies in the fact that empirical science cannot (yet) satisfyingly differentiate between neural states that are consciously experienced and neural states that are happening unconsciously. Simply said, neural conditions necessary for a neural state to break from unconsciousness and become part of the conscious experience are still unknown. This is actually nothing new. It is essentially only a rephrasing of the most important question that has concerned neuroscientists and neurophilosophers since the conscious experience became a topic of empirical science: what are “(…) the minimal sets of neuronal events that give rise to a specific aspect of a conscious percept?” (Crick and Koch 2003).

The current state of the art of empirical studies of consciousness is nowhere near to answering this fundamental question, and the search for a “minimal set of neural events” that would capture the crucial distinction between consciousness and unconsciousness processing seems far from over.

### State of the art

Conscious experience was originally purely a philosophical topic considered too mysterious for empirical science. This field was dominated by highly abstract questions, such as Is it possible to reduce subjective consciousness to mere material brain states (Armstrong 1968)? If consciousness could be reduced to material brain states, would it be ethical to manipulate it (Metzinger 2009)? Is consciousness an emergent property of the brain (Searle and Brown 1984)? What is the relationship between consciousness and free will (Libet 1999)? Is there really a hard problem of consciousness (Chalmers 1996), claiming that neuroscience (based on physicalism) will never be able to explain why and how are physical processes between neurons accompanied by phenomenal conscious experience?

The history of empirical research of consciousness (see LeDoux et al. 2020 for a detailed review) is closely tied to unconscious neural processes related to blindsight (Pöppel et al. 1973, Sanders et al. 1974, Weiskrantz et al. 1974) and split-brain (Bogen 1962, Gazzaniga et al. 1962, Gazzaniga 1985, Gazzaniga and LeDoux 2013). In both cases, patients responded and appropriately categorized presented stimuli but repeatedly claimed to not consciously experience them. Such behaviour emphasized the difference between unconscious and conscious processing in the brain and laid the groundwork for empirical studies of consciousness. Years later, Francis Crick and Gerald Edelman presented their interpretations of consciousness as an empirical problem along with neural processes responsible for the emergence of conscious experience. Francis Crick considered gamma synchronization as the marker and the main correlate of conscious experience (Crick and Koch 1990), while Gerald Edelman claimed conscious experience is a result of thalamocortical re-entrant loops (Edelman 1989, 2004).

The current research of consciousness occupies a vaguely defined and blurred line between empirical science and neurophilosophy. Many tools for its research have been developed, ranging from highly abstract and speculative thought experiments with bats (Nagel 1974), colourless rooms (Jackson 1982), and zombie doppelgangers (Chalmers 1996), to specifically developed experimental conditions, such as pattern illusion paradigms (Harris et al. 2011); various kinds of visual illusions (Kirschfeld 1999); attentional paradigms (Simons and Chabris 1999); masking paradigms (Bachmann and Francis 2013); multistable paradigms, such as binocular rivalry (Blake et al. 2014), flash suppression (Wilke et al. 2003), and its modern variants continuous flash suppression (Tsuchiya and Koch 2004); and breaking continuous flash suppression (Yang et al. 2007). Nowadays, paradigms for studying other modalities of perceptual consciousness, such as auditory consciousness (Brancucci et al. 2011) and olfactory consciousness (Gottfried 2009, Mori et al. 2013), are being tested.

Even though the empirical research of consciousness is now established as a full-fledged empirical topic, its current state of the art seems to be stuck between the ceaseless proliferation of theories of consciousness (Table 1), revealing its own limits, shortcomings, and knowledge gaps (Table 2) rather than providing minimal sets of causal mechanisms of conscious experience. The main reason for this current state of the art has been the emphasis on observational methodology, which sets as its main objective the revealing of the neural correlates of consciousness (NCCs).

**Table 1.** Theories of consciousness

Higher-Order Theory of Consciousness (Lau and Rosenthal 2011)	The core of this theory lies in higher-order representations that target lower-order representations. The difference between unconscious and conscious content is based on its meta-representation by higher-order mechanisms.
Attended Intermediate Representation Theory (Prinz 2012)	Contents of experience are associated with intermediate representations. Such content becomes part of conscious experience when it becomes available to working memory through attention that is associated with gamma synchrony.
First-Order Representation Theory (Mehta and Mashour 2013)	Contents of experience and their phenomenal character, addressed as specific consciousness, are associated with sensory regions. On the other hand, post-sensory regions are associated with general consciousness, which makes content conscious.
Recurrent Processing Theory (Lamme 2006, Lamme 2010)	Consciousness is associated with recurrent or re-entrant processing, which occurs in highly interconnected sensory systems with the involvement of feedforward and feedback connections.
Global Neuronal Workspace Theory (Dehaene 2014)	Consciousness is considered as a global neuronal workspace that consists of prefronto-parietal brain regions. The entry of content into the stream of consciousness lies in ignition triggered by bottom-up sensory activation and top-down amplification of the sensory signals.
Posterior Cortical Hot Zone Theory (Koch et al. 2016)	This theory associates consciousness with local activations within posterior parts of the brain, namely parietal, temporal, and occipital lobes, while leaving the frontal cortex out of the consciousness processing.

Several theories of consciousness have been developed over the years. However, many of them are closer to metaphysics than neurobiology. Therefore, this table briefly describes only those that are closest to neurobiology, and their key points are empirically testable.

**Table 2.** Knowledge gaps

“Distinction gaps” point out the difficulty of making a clear distinction between conscious processing, unconscious processing, other types of consciousness, and other accompanying cognitive states. For example:
i. Is it possible to satisfactorily distinguish between unconscious prerequisites, genuine consciousness experience, and unconscious consequences (Aru et al. 2012)?
ii. Is it possible to satisfactorily distinguish between genuine conscious experience and meta-consciousness (Frässle et al. 2014)?
iii. Is it even possible to distinguish between full NCCs that represent experiences in general, content-specific NCCs, such as representations of faces, and background states of NCCs, such as alertness (Koch et al. 2016)?
iv. Is the attention a necessary part of the conscious experience or not, and if so, can we distinguish it from consciousness (Koch and Tsuchiya 2007; Prinz 2012)?
“Pollution gaps” apply to experimental endeavours in which actively reporting one’s subjective experiences is an integral part of experimental measurements. For example:
i. Does active reporting during experiments pollute the measured data and prevent finding genuine correlates of conscious experience (Tsuchiya et al. 2015)?
ii. Will we ever find behavioural markers that would replace active reporting (Koch et al. 2016)?
“Universality gaps” is a set of questions that considers the universality of correlates among the different (sensory or intrinsic) modalities of consciousness. For example:
i. Can we consider the NCCs of visual consciousness as universal NCCs? What are the NCCs of the other domains of conscious experience, such as olfactory consciousness (Mori et al. 2013), auditory consciousness (Steinmann et al. 2014), and tactile consciousness (Meador et al. 2002), and are they in some way identical to visual NCCs?
“Minimal sets gaps” summarize together all the questions of what the minimally sufficient and necessary conditions are for representations to become part of the conscious experience. For example:
i. Is the activity of a specific sensory area the only thing sufficient and necessary for the specific representation/content to become conscious (Block 2009; Zeki 2007)?
ii. Or is the coactivity of large-scale brain areas necessary (Dehaene 2014; Dehaene et al. 2003)?
iii. Are the frontal regions of the brain necessary for conscious experience, and if so, what is their role (Boly et al. 2017)?

Knowledge gaps within conscious studies may be roughly divided into several categories, such as “distinction gaps”, “pollution gaps”, “universality gaps”, and “minimal sets gaps”. The number of described gaps is in no way final, and the proposed categories do not have strict boundaries that would prevent one topic to pass to another category.

### Neural correlates of consciousness

NCCs are the building blocks of empirical studies of consciousness. They represent the minimal observable sets of neuronal events that are necessary for specific conscious percept (Crick and Koch 2003, Koch et al. 2016) and for the distinction between conscious and unconscious processing. Thorough theoretical and philosophical work on NCCs has been performed (Metzinger 2000), and many NCCs were proposed, starting first from specific brain areas, such as the thalamus (Penfield 1938) primary visual cortex, or specific local activations of parietal brain areas (Koch et al. 2016), through specific brain rhythms and their synchronization, such as P300 (Babiloni et al. 2006, Del Cul et al. 2007, Lamy et al. 2009, Dehaene and Changeux 2011, Salti et al. 2012, King et al. 2014) or gamma synchrony (Crick and Koch 1990, Llinas et al. 1994, Engel et al. 1999), connectivity, such as thalamocortical re-entrant loops (Edelman 1989, 2004), or connectivity of frontoparietal brain areas through parts of the thalamus (Dehaene 2014), to their various combinations in theories of consciousness, such as Attended Intermediate Representation Theory (Prinz 2012), Posterior Cortical Hot Zone Theory (Koch et al. 2016), Global Neuronal Workspace Theory (Dehaene 2014), and many others. To date, no specific correlate or theory has been accepted as universal, sufficient, or satisfactory. In consequence, the current studies of consciousness turn towards explaining consciousness within tenets of other theories, such as predictive processing theory (Havlík et al. 2017, Marvan and Havlík 2021), as if a universal theory of brain processing (that is still speculative and unproved) should provide the missing link to finding NCCs. We conjecture that the main reason for this current state of the art lies most likely in the methodological limitations of the correlational approach.

#### Methodological limitations of the correlational approach

The main body of research of conscious experience is based on observations of time series of neural activity or events typically obtained by neuroimaging techniques, such as functional magnetic resonance imaging (fMRI), electroencephalography (EEG), positron emission tomography, or magnetoencephalography. Neurophysiological correlates may, in principle, also be registered by brain recording by intracortical microelectrodes or microdialysis used to directly identify the changes in brain activity or chemistry. Even though the first two (fMRI and EEG) are the most commonly used for the research of consciousness, all of the mentioned methods encounter the same limitations. Research conducted in this way is methodologically correct but principally correlative in nature; therefore, it only reports that two events occur together, either at the same time or one after other, but strictly without any causal relationship. It is possible to assume that there is a causal relationship, but it cannot be proven or disproven on the basis of a correlational approach.

However, without establishing the causal nature of the relation between the two events, it is quite difficult to establish the minimal sets of neural conditions necessary for specific content to become part of consciousness.

On the one hand, this claim is trivial and mostly agreed upon in empirical studies of consciousness. On the other hand, it is still the most pressing problem in the contemporary empirical science of consciousness. There is a difference between the correlational distinction and the causal distinction between conscious and unconscious processing.

To distinguish conscious processing from unconscious processing, empirical studies of consciousness (based on correlational methodology) have utilized the so-called “contrasting condition” (Baars 1993) or “contrastive method” (Aru et al. 2012) from early time. This method lies in the contrast between two conditions, one happening with conscious experience and the other without conscious experience. The contrast between these two conditions should in theory reveal the brain region or activity that would be considered as the reliable NCC and the minimal set of neural conditions necessary for consciousness. Even though contrast analysis and correlational research may methodologically show the neural conditions that may help distinguish between conscious and unconscious processing, such a distinction is still missing something crucial. The observed brain activity cannot be considered as proof that brain activity causes the neural content to be conscious. Observing the difference between conscious and unconscious processing via the contrast between two conditions is something different from providing minimal sets of neural events that are causally effective for specific content to surpass unconscious processing and become part of the conscious experience. Put simply, finding the difference between unconscious brain processing and conscious brain processing is not the same as finding the specific mechanisms that are responsible for this “switch”. One may find a specific recurring set of neural conditions that perfectly identify a conscious state from an unconscious state (by correlational methodology) and still not know whether it is causally linked to the conscious state or only its correlate (with another common cause or even reverted causality: consciousness affecting the brain states). In other words, knowing correlates is not knowing the cause–effect chain, at least not in principle! In order to find reliable minimal sets of neural events that are responsible for content to become conscious, a causal explanation is needed.

A counter-argument may immediately be raised claiming that most of the empirical experiments deal with causality in the form of manipulation with stimuli. True, creating specific conditions and manipulation with stimuli is the “daily bread” of perceptual (visual) studies of consciousness. For example, during the binocular rivalry paradigm (Klink et al. 2013), two different stimuli (e.g. a house and a face) are presented to each eye separately. Representations of these stimuli remain the same on the neural level but spontaneously fluctuate between conscious and unconscious processing. Without this manipulation, the consequent comparison of conscious and unconscious states of specific representation would be virtually impossible, and the only comparison that could be performed would be a state of vast visual conscious experience with a state without conscious experience—such as a coma or deep sleep. Such a comparison would not bring any usable data about specific visual content and its conscious/unconscious processing.

Causal manipulation with visual inputs is a methodological necessity that narrows down the visual conscious experience. Some causality in the form of manipulation is present, but what is manipulated is the sensory input not the brain state directly. However, if it would be possible to precisely manipulate the brain through manipulation with stimuli, this would effectively become manipulating the brain and would be an experiment we would be happy with. However, control over brain states by stimulus manipulation is limited, which leads to only imperfect experiments, asymptotically limited to pure correlational research as the methods become more and more clumsy with observational methods, such as fMRI and EEG. In these cases, sensory manipulation is insufficient for reliable causal alteration of the brain. Again, this form of stimulus manipulation is necessary for observation and subsequent utilization of a contrastive method but not sufficient for revealing the causal mechanisms of consciousness. Therefore, our argument stands.

Another limitation of the correlational methodology is that it cannot provide a definite answer to the functional relevance of a specific neural region in conscious experience. The observed activity may only be an epiphenomenon, or it may be linked to different brain functions, completely unrelated to conscious experience, such as the activity of the motor cortex, cerebellum, or possibly the frontal brain regions (Safavi et al. 2014). Frontal brain areas are generally reported in experiments of consciousness and also represent an integral part of the theories of consciousness (Lau and Rosenthal 2011, Dehaene 2014). However, their relevance to conscious experience is still a matter of ongoing discussion (see Boly et al. 2017 and Odegaard et al. 2017 for a persistent disagreement). Frässle et al. (2014) came to the conclusion that frontal areas are not necessary for conscious experience but are generally involved in other processes, such as monitoring of conscious content, introspection, and active reports. Furthermore, there are suggestions that frontal brain areas are responsible for top-down control of perceptual switches during binocular rivalry (Wilcke et al. 2009, Britz and Pitts 2011, Britz et al. 2011, Buckthought et al. 2011, Knapen et al. 2011, Pitts and Britz 2011). But are these switches part of the NCCs, do they belong to completely different brain operations, or are they only observable epiphenomena that occur on the unconscious level?

In this way, the correlational methodology is not only unable to reveal reliable mechanisms responsible for the content to enter conscious experience (first limitation) but also cannot reveal the functional relevance of observable brain regions. Due to this, the correlational methodology may unintentionally confound correlates of consciousness with other supportive brain functions or other brain epiphenomena. This is in no way intentional, but it still represents yet another limitation that in turn may be severely misleading for research of consciousness. As opposed to correlates, the mechanisms that bring about effects from causes require a causal explanation between neural states and consciousness together with an explanation of the functional relevance of a specific mechanism.

The third and final limitation is also connected to confounding but not to correlates with epiphenomena or other functions but various mechanisms together. Let’s consider there is not one mechanism responsible for the content to become conscious but several functionally distinct neural mechanisms of consciousness. The question is, Is the correlative approach capable of observing and describing such interconnected but functionally different mechanisms that together create a coherent conscious experience?

Correlational methodology based on neuroimaging provides images of “blobs” (known as “blobology”) of active brain areas (Poldrack 2012), specific frequency and spikes (Babiloni et al. 2006, Doesburg et al. 2009), or measures of functional dependence of a more than one time series of biosignal with connectivity as a typical example.

However, it cannot go any further.

Observation of “blobs” of brain activity cannot determine whether one or many mechanisms of consciousness are present, and it also cannot reveal their different functions. Using correlational methodology cannot give a clear understanding of the functional difference between specific areas of the brain and what roles they play in the emergence of conscious experience. Such research will always be uncertain about the actual underlying neural mechanisms that cause content to become part of conscious experience because the correlational methodology does not allow these hypothetical mechanisms to be tested separately.

The idea of various functionally distinct mechanisms within conscious experience may be traced in the literature. For example, the largely neglected theory of First-Order Representationalism pointed out several gaps in other theories (Mehta and Mashour 2013), mainly the specific and distinct functional roles of post-sensory and sensory regions (Mehta and Mashour 2013). According to this theory, sensory regions are responsible for specific consciousness, which gives a state its specific phenomenal character, while post-sensory regions are associated with general consciousness, which makes a state conscious. This theory bears resemblance to Lau’s and Rosenthal’s Higher Order Theory (Lau and Rosenthal 2011), in which the authors also consider different functions of sensory and post-sensory regions. First-order representations depend on sensory regions, while higher-order representations that are necessary for conscious awareness are associated with prefrontal and parietal brain areas. Furthermore, there are several neglected regions whose functions are very interesting in terms of consciousness, such as insular regions (Seeley et al. 2007, Corbetta et al. 2008), which are rarely seen in theories of consciousness or NCC reports.

Neglecting and confounding distinct mechanisms of consciousness is understandable. On the other hand, it is not possible to ignore this issue for long because it may be a fundamental knowledge gap within the current studies of consciousness. Observational research does not allow to test separately, disentangle, and thereby prove the existence of functionally distinct neural mechanisms of conscious experience. However, in order to progress, the science of consciousness needs to reconsider its own methodology, which is still based largely on a correlational approach. The solution to the above-mentioned limitations as well as the description of the mechanisms of consciousness may lie to some extend in the methodology of causal explanation.

#### In the defence of correlational methodology

The three main limitations of the correlation methodology have been described earlier. Although at first glance it might appear that research of consciousness has succumbed to the correlational fallacy, this is not the case. The above-mentioned sections should not give the impression that the correlational approach is wrong, should be abandoned, or cannot provide anything about consciousness. The correlational approach is necessary to establish hypotheses and narrow down potential candidates for further rigorous causal experiments. Moreover, even causal research on consciousness is not entirely immune from the criticisms outlined earlier. For example, lesion studies can and should be considered as part of causal studies of consciousness, but while some lesions have shown clear deficits in conscious experience (see the section on “Evidence for the three distinct mechanisms of consciousness”), some are still subject to debate, particularly, but not exclusively, the role of the prefrontal cortex in conscious experience (see Boly et al. 2017 and Odegaard et al. 2017). Simple lesions or deactivation of separate brain regions cannot be considered sufficient evidence to clearly describe their role in conscious experience. Various regions can be responsible for a number of other neural functions, and their loss can obscure conscious experience or create conditions that completely invalidate any possibility of inquiring consciousness.

Nevertheless, the methodology of causal explanation through causal alterations remains the best viable option for subsequent research on consciousness. It must be based on spatially precise stimulation, reversibility of alterations (not irreversible lesions), and bidirectional causal alterations (see the section on “Mechanisms, causal explanation, and causal alterations”). Carefully prepared experiments have to take into account connections between specific brain regions and their different responses towards inhibition and excitation. This is the only way to describe and explain the underlying neural mechanisms necessary for the emergence of consciousness.

### Mechanisms, causal explanation, and causal alterations

Nothing is more natural for the human mind than reasoning in causal models. Inferring the states of the world as effects of causes is a common reasoning strategy next to inductive and abductive reasoning. According to Sloman (2005), humans utilize causal models for predictions, expectations, learning, reviewing past decisions, and of course actions—past, present, and future. Humans spend ∼50% of their day in mind-wandering episodes (Killingsworth and Gilbert 2010, Song and Wang 2012, Havlík 2017, Havlík et al. 2019). Many of these episodes probably have the form of various hypothetical causal models with different causes and effects that describe the different possible worlds. Consequently, it is a common and intuitive strategy to develop several possible worlds (predictions) and choose the best possible one while sacrificing others (Dennett 2008). Moreover, it is possible to consider that the unity of conscious reasoning is holding together, thanks to thinking in the form of cause–effect chains or causal trees. Reasoning in causal models is as natural for humans as their inner understanding of laws of causation: causes precede effects and not the other way around; if sufficient causes do not occur, the predicted effects also do not occur; and the changes in effects cannot change causes.

The difference between correlates and mechanisms lies in causal models and causal explanation. Again, correlates denote events in the world that co-occur together, one happens if and only if the other happens too, without any causal explanation that the second event is the effect of the first event (cause). The influential theorists of neuroscience (Craver 2007, Bickle 2008, Bechtel and Richardson 2010, Piccinini 2020) have assumed that the understanding and explanation of complex phenomena or behaviour of a complex system (such as the brain) are achievable only through a thorough understanding of the specific functional parts, their interaction, and the causal roles they play within the system. This is called the “mechanistic explanation”, which does not focus on correlation but directly on causal mechanisms governing the mutual interactions between the individual parts of the complex system.

To speak about mechanisms is to speak about explaining causal events which, however, entails a number of obligations (Sloman 2005), which correlational research does not have to fulfil. At first, it is necessary to have at least some basic knowledge about which inputs (causes) may produce specific outputs (effects) within a complex system (such as the brain). Another obligation of causal explanation is known as “counterfactual dependence”. This obligation says that if the cause (input) does not happen, then the effect (output) will also not happen. Finally, there is the obligation of causal alteration (manipulation), which is crucial for any experiment. Causal alteration is the manipulation of an independent variable (cause) that has to have an effect on a dependent variable (effect). Causal alterations (various “do operations”, activation, excitations, and inhibitions) enhance or abolish the observable effects if there is a causal relation between them.

The core of the mechanistic explanation consists of “decomposition” and “localization” (Bickle 2008, Bechtel and Richardson 2010). Considering consciousness as a property of the brain that consists of and is identical to specific brain mechanisms, “decomposition” is a necessary research strategy when a top-down causal explanation is required. Top-down causal explanations start with the phenomenon we want to explain—consciousness. Its explanation requires the gradual decomposition of the complex system (brain) into components. The system consists of an unspecified number of levels as it may be divided into subsystems, subsystems into parts, and specific parts into subparts, and so on (brain → lobes, lobes → brain regions or gyri, regions → neurons, neurons → synapses, synapses (and neurotransmitters) → intracellular computations, etc.). However, due to the limits of our capacity, it is necessary to choose only several components for a mechanistic explanation. Brains are too complex machines, with a large number of components, levels, and their complex interactions, to be computationally tractable. The same applies to the selection of level(s), such as intracellular computations where the choice is limited by current technology. Therefore, it is necessary to focus only on a few subsystems at reachable levels in a complex system before subsequent decomposition. For example, it is reasonable to first consider the causal interaction of higher levels (brain regions) in an explanation of consciousness before focusing on the lower levels (synapses, neurons, proteins, etc.). “Localization” focuses on the identification of individual mechanisms and their functions in decomposed components, such as the specific function of a specific brain region or the specific function of a specific neuronal population at lower levels. Localization focuses primarily on identifying the so-called locus of control, which is the main source of a specific observed behaviour of the system (Libet 1999).

Causal alterations of specific components (the so-called “do operation” in causal modelling) are a necessary part of the mechanistic explanation. They are the only thing that enables inferences in causal models and an explanation of phenomena per se. There are two basic methods of causal alteration—“inhibition” and “excitation” (Bickle 2008, Bechtel and Richardson 2010). In the case of inhibition, a specific part of the system is withdrawn from the causal structure of the complex system, leading to the subsequent inhibition or even abolition of the observable phenomenon or system’s behaviour (if the system part and phenomena are causally connected). This analytical method was applied to early studies of cerebral localization based on direct invasive experiments, resulting in severe cognitive or behavioural deficits. Examples of early cases of inhibition experiments include invasive experiments on animals, such as the destruction of the amygdala in rhesus macaques resulting in the loss of aggressive behaviour (Purves et al. 2004); lesions of speech centres resulting in receptive and motor aphasia discovered by Broca and Wernicke (Purves et al. 2004); inhibition of the posterior parietal cortex (PPC) of cats using an electric current that induced significant online decreases in correct detection and localization of targets presented in the contralateral visual hemifield (Schweid et al. 2008); or the inhibition of the cAMP response element-binding protein in rodents that resulted in the abolition of new memories whereby indicating its importance in the formation of long-term memories (Viola et al. 2000, Bickle 2008). Excitation, on the other hand, facilitates or triggers the activity of specific parts within the complex system and amplifies the concrete observable phenomenon or system’s behaviour (if both belong to the same causal structure). In this case, simple examples may be given as direct electric stimulation of e.g. the motor cortex, in which automatic responses occur in the form of observable motor responses or induce changes of excitability in motor-evoked potentials (Nitsche and Paulus 2000, 2001, Nitsche et al. 2003). On a higher level, the stimulation of prefrontal areas leads to an enhancement of working memory (Fregni et al. 2005), and it may also modulate speech perception, support perceptual learning (Rufener et al. 2016), and enhance facial emotion perception (Janik et al. 2015).

These represent only a fraction of previously performed stimulation studies, but they clearly demonstrate two important facts. First, there is a causal link between brain stimulation and altered states of mind. Second, analytical methods of inhibition and excitation work on various levels ranging from big brain areas to cell biology and are therefore universal methods for revealing the functional relevance of mechanisms and their causal role within a complex system. Note that while the distinction between inhibition and excitation describes the key abstract types of possible experimental manipulations, in practice manipulation by the resection of experimental brain stimulation at one level may have a combined effect due to the complex structure of the affected subparts. For instance, the resected area contains both excitatory and inhibitory neurons with different projections to other areas, and therefore, its resection may have a complex effect on the remaining areas.

Describing the causal mechanisms of the complex system via causal alterations does not share the methodological limitations of the correlational approach described in the previous section.

The contrasting condition utilized by the correlational approach allows in principle to distinguish between conscious and unconscious processing. However, such a distinction may always be called into question on the grounds that even perfectly identified correlates are unreliable because of a missing causal relationship and causal explanation. On the other hand, causal changes in specific neural mechanisms not only make it possible to distinguish between conscious and unconscious processing but also make it possible to find the minimal sets of neural mechanisms that are directly responsible for making specific content part of conscious experience. Establishing such a causal relationship is methodologically much stronger and more reliable than identifying NCCs.

The second limitation of the correlational approach is described as the inability to determine the functional relevance of the observed active brain region. Based on observation alone, it is impossible to determine whether specific brain activity correlates with conscious processing or with other unrelated brain functions. Causal alterations in the form of “do operations” (excitation and inhibition) also overcome this limitation. Utilizing these methods allows to experimentally decide whether the observed activity has a causal influence on conscious experience or whether it is only an observed epiphenomenon whose causal alteration will not have any causal effect on consciousness.

Finally, the third limitation of the correlation approach is its inability to resolve whether there is one mechanism of consciousness or several different mechanisms. This distinction cannot be made by observing neural “blobs” alone. The methodology of causal alterations may also be utilized to overcome this limitation. Excitation and inhibition together may reveal the functionally different mechanisms of consciousness and disentangle them from each other with a series of experiments. If the conscious percept is abolished or inhibited by manipulating specific brain regions, then it is possible to describe the functional role of a specific mechanism (brain region or specific frequency) and separate its role from other mechanisms.

Revealing functionally distinct mechanisms of consciousness via causal alterations should be the next step in the science of consciousness that will move away from uncertain NCCs to actual mechanisms and their causal interactions. Therefore, observational research needs to be supplemented by neurostimulation methods (enabling both excitatory and inhibitory “do operations”), such as transcranial magnetic stimulation (TMS), transcranial electric stimulation (TES), and, the new tool for the job, geodesic transcranial electric neuromodulation (GTEN). Importantly, while neurostimulation methods have poor observational possibilities next to observational methods, they are able to overcome the main methodological limitations of correlational methodology.

## Brain stimulation methods in consciousness research

There are currently several non-invasive brain stimulation tools that have been utilized during the research of consciousness, namely, TMS and TES.

TMS equipment consists of a coil linked to a strong capacitator. A brief electric current leads to a discharge of a magnetic field, which is delivered to the underlying brain cells causing action potentials (Wassermann et al. 2008). TMS may be applied in several forms: single pulses, two paired pulses, and repetitive TMS applied in a series of pulses of varying frequencies (Ekhtiari et al. 2019). Consequently, the TMS causes reversible changes in brain processing, and its effects may be observed in the form of motoric responses (Pascual-Leone et al. 1994b), reported perceptions (Horacek et al. 2007, Klirova et al. 2013), such as phosphenes (Schaeffner and Welchman 2017), cognitive tasks (Martin et al. 2017), emotions (Prasko et al. 2006, 2007, Horacek et al. 2015, Kazemi et al. 2016), and memory (Skrdlantova et al. 2005, Beynel et al. 2019). On the other hand, repetitive TMS may interfere with neuronal processing via “virtual lesions” (Weissman-Fogel and Granovsky 2019) and disrupt perceptual (Amassian et al. 1989, Pascual-Leone et al. 2000) and also cognitive processing (Sack et al. 2002a, 2002b). However, TMS is insufficient for more selective targeting of deeper brain structures. The standard range of the magnetic coil is 2 cm, and TMS coils with a deeper range of magnetic field designed for deep TMS influence large brain areas non-selectively.

Compared to TMS, TES methods work on the principle of administering a weak electric current to a selected brain area in which cortical excitability is modulated as a result of electric stimulation. The main difference between TES and TMS is that TES does not evoke action potential (Radman et al. 2009, Reed and Cohen Kadosh 2018). This is due to the weak electric current of the stimulation (not exceeding 1–2 mA) as higher dosages become uncomfortable for human participants. However, although TES is not evoking action potential, it is still sufficient for causal alterations of underlying neuronal tissue. According to Klírová (2022) (Nitsche and Paulus 2011, Paulus 2011), TES affects the resting potential of neurons, and it may either depolarize or hyperpolarize the membrane, whereby increasing or reducing the neuron’s firing rate (Creutzfeldt et al. 1962, Purpura and McMurtry 1965). TES affects Gamma-Aminobutyric Acid and glutamate levels (Stagg et al. 2009), and it is hypothesized that through tonic depolarization of axon terminals, TES leads to the release of neurotransmitters without generating action potentials (Liu et al. 2018). The effects of TES are also associated with the repetitive opening of Na^+^ channels (Schoen and Fromherz 2008, Antal and Herrmann 2016), with a possible increase in synaptic transmission by enhancement of intracellular concentrations of Ca^2+^ in astrocytes (Monai et al. 2016), and strengthening of glutamatergic synapses (Fregni et al. 2005). As with TMS, TES methods also have their limitations. For example, the skull and skin tend to obstruct TES, resulting in a relatively limited amount of current that reaches the cortex (Liu et al. 2018, Vöröslakos et al. 2018). Also, TES methods are not able to selectively target deeper brain structures. This represents a major limitation for studies of consciousness, as many key regions of consciousness are located in the deeper structures of the brain.

Intracranial electric stimulation is occasionally used for studying conscious experience as part of the presurgical evaluation (Parvizi et al. 2012, Foster and Parvizi 2017, Fox et al. 2018). Its specific type, deep brain stimulation has no problem of reaching deep parts of the brain. However, this method is highly invasive and can only be used to study the conscious experience of patients. Both optogenetics (Chen et al. 2021) and low-intensity focused ultrasound (Arulpragasam et al. 2022) are considered anatomically precise stimulation tools that are able to stimulate deep brain structures, but their current use in the studies of consciousness is sporadic. However, in this review, we will focus on recently developed GTEN.

## Geodesic transcranial electric neuromodulation—a tool for anatomically precise causal alterations in brain

GTEN is a promising new approach to studying conscious experience. GTEN addresses many of the limitations of other stimulation methods, including TES, by offering a high degree of anatomic precision and the ability to target large-scale brain networks or specific brain regions, including deep structures. Additionally, GTEN is non-invasive and relatively inexpensive.

GTEN enables the so-called multielectrode transcranial electric stimulation, which optimizes the required current density in the targeted brain area. Multielectrode transcranial electric stimulation analyses (Dmochowski et al. 2011, Fernandez-Corazza et al. 2016) have shown that the use of a higher electrode density improves focus, directionality, and stimulation intensity parameters by penetrating the deeper brain structures. Furthermore, GTEN also meets (to some extent) the needs of the future generation of TES technology described by Liu et al. (2018): “(I) delivery of stronger currents to the brain while minimizing peripheral and indirect effects, (II) simultaneous stimulation and recording of brain activity for quantitative measurements of TES-induced effects, and (III) targeting of specific rhythms through closed-loop stimulation of brain areas, including deep brain structures”.

Considering the first point, GTEN applies a weak electric current (maximum of 2 mA) that is still considered “gentle” and is frequently used on healthy participants (Liu et al. 2018). In this regard, GTEN behaves as a classical TES brain-modulating device; however, GTEN is assembled from 256 electrodes for current stimulation (and possible EEG monitoring). Multi-electric high-definition TES using computational modelling based on individual magnetic resonance imaging (MRI) is considered one possible way of providing more focal stimulation (Datta et al. 2009, Edwards et al. 2013) and enhancing the current density at the intended target area (Liu et al. 2018). Each of the 256 electrodes is able to act as an anode or cathode for transcranial direct-current stimulation (tDSC) protocol (see the next section) and is optimized for purposes of the strongest transmembrane polarization that is expected as the electric fields overlap inside the brain (Liu et al. 2018). To minimize peripheral and indirect effects, GTEN uses artificial or real head models obtained from MRI. Co-registration of the individual Global Positioning System locations of the electrodes positioned on the scalp with individual MRI allows for morphologically precise stimulation of brain areas (the so-called “model planning”), considering individual head and brain anatomy together with cortical geometry. In other words, GTEN modulates the functioning of the desired parts of the brain with very high selectivity, which is further enhanced by using real head-brain models unique to each participant (Liu et al. 2018, Klírová et al. 2021). While GTEN cannot apply a stronger current, it is able to build up a stronger current density and increase focality without increasing the unwanted peripheral stimulation (Datta et al. 2009, Dmochowski et al. 2011, Liu et al. 2018). In short, GTEN maximizes the current targeting accuracy and minimizes off-target activation within the rest of the brain.

Considering the second point, GTEN meets the condition of simultaneous stimulation and recording of brain activity halfway. GTEN is capable of simultaneous current stimulation and EEG monitoring; however, the electrodes used for stimulation cannot be used simultaneously for monitoring. The question is how precise the EEG recording will be and how many artefacts will appear in the recording due to concurrent current stimulation. This applies to online stimulation experiments, i.e. simultaneous current stimulation and EEG monitoring. Another possibility is to use offline stimulation experiments, where EEG monitoring takes place after the stimulation. In offline experiments, GTEN excels in monitoring EEG activity altered by the stimulation because the caps used for GTEN are identical to the state-of-the-art high-density 256-channel EEG caps. Therefore, immediately after the stimulation, the very same cap may be used for EEG monitoring. Conveniently, the neuromodulation effects last long after the end of the stimulation, lasting up to 1–1.5 hours (Terney et al. 2008), and EEG changes in cortical excitability may be contrasted with a sham stimulation (control condition) after the end of stimulation.

**Figure 1.**
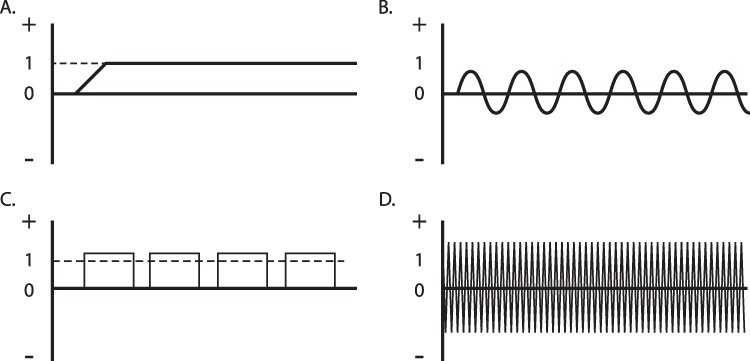
TES protocols

Considering the third point, GTEN has access to all standard TES protocols (Fig. 1) such as transcranial direct current stimulation (tDCS), transcranial alternating current stimulation (tACS), transcranial pulsed current stimulation (tPCS), and transcranial random noise stimulation (tRNS). Targeting specific brain rhythms is relatively easy and may be performed by tACS where any desired frequency may be used. Furthermore, GTEN is also capable of stimulating different brain regions simultaneously. Therefore, it is able to create various frequency co-activations between distant brain regions. Targeting deep brain structures is usually a problem for standard TES tools and protocols.

Several protocols of transcranial electric stimulation have been developed over the years. A) Transcranial direct-current stimulation (tDCS) was the first to be developed and is the most commonly used. tDCS utilises a direct electric current, the unchanging flow between two electrodes – an anode and a cathode. B) Transcranial alternating current stimulation (tACS) (Antal et al. 2008; Bächinger et al. 2017; Herrmann et al. 2013; Inukai et al. 2016; Weinrich et al. 2017) relies on changes in regular sinusoidal ups and downs that are characteristic of an alternating current. The tACS protocol is not polarity dependent like tDCS, but is based on frequency; therefore, its causal effects on neural structures do not depend on anodal and cathodal stimulation but are elicited by the amplitude, frequency of the current, and orientation of the penetrating current (Polanía et al. 2018; Radman et al. 2009). C) Transcranial pulsed current stimulation (tPCS) (Morales-Quezada et al. 2015; Morales-Quezada et al. 2014; Vasquez et al. 2016) has recently been developed as a special form of direct current stimulation (Jaberzadeh et al. 2014; Jaberzadeh et al. 2015). It is based not on a continuous current, but pulses are delivered in specific ranges of milliseconds (500 ms; 250 ms; etc.) separated from each other by a “rest” (non-stimulation) that usually lasts tens of milliseconds. D) Transcranial random noise stimulation (tRNS) is a modification of tACS that does not rely on a specific frequency. Its frequency and intensity of current are randomised within a range of 0.1 to 640 Hz (Antal and Herrmann, 2016; Terney et al. 2008).

An individual head computational model optimizes electrode placement and current intensity at a given electrode with the required current density in a selected brain region, including deeper cortical regions such as the anterior cingulate cortex (Klírová et al. 2021). It was confirmed that high-definition transcranial electrical stimulation at low intensities and with small electrodes can generate electric fields in deep brain structures, depending on the stimulation intensity and electrode montage (Louviot et al. 2022). Also, GTEN is able to utilize the tPCS that is supposed to even reach structures such as the insula, thalamus, and hypothalamus (Datta et al. 2013). GTEN utilization of MRI and individual brain anatomy also allows deep brain structures to be reached much better and more reliably than standard electric stimulation tools.

Several protocols of TES have been developed over the years. (I) tDCS was the first to be developed and is the most commonly used. tDCS utilizes a direct electric current and the unchanging flow between two electrodes—an anode and a cathode. (II) tACS (Antal et al. 2008, Herrmann et al. 2013, Inukai et al. 2016, Bächinger et al. 2017, Weinrich et al. 2017) relies on changes in regular sinusoidal ups and downs that are characteristic of an alternating current. The tACS protocol is not polarity dependent like tDCS but is based on frequency; therefore, its causal effects on neural structures do not depend on anodal and cathodal stimulation but are elicited by the amplitude, frequency of the current, and orientation of the penetrating current (Radman et al. 2009, Polanía et al. 2018). (III) tPCS (Morales-Quezada et al. 2014, 2015, Vasquez et al. 2016) has recently been developed as a special form of direct current stimulation (Jaberzadeh et al. 2014, 2015). It is based not on a continuous current, but pulses are delivered in specific ranges of milliseconds (500 milliseconds, 250 milliseconds, etc.) separated from each other by a “rest” (non-stimulation) that usually lasts tens of milliseconds. (IV) tRNS is a modification of tACS that does not rely on a specific frequency. Its frequency and intensity of current are randomized within a range of 0.1–640 Hz (Terney et al. 2008, Antal and Herrmann 2016).

### TES protocols as tools of causal alterations and their comparison

TES protocols have a direct influence on cortical excitability and may therefore be utilized for causal alterations, excitation, and inhibition. The tDCS protocol may be used for either excitation or inhibition by utilizing anodal or cathodal stimulation. Anodal stimulation results in an increase in neural excitability, while cathodal stimulation results in a decrease in neural excitability (Nitsche and Paulus 2000, 2001, Rogalewski et al. 2004, Fregni et al. 2005, Schweid et al. 2008, Karabanov et al. 2019). The tACS excitation and inhibition effects are based on applying a frequency to a specific underlying cortical area. If the cortical tissue is stimulated with a current of a frequency lower than the intrinsic frequency of the neural structure, then neural oscillations will be inhibited (Fröhlich and McCormick 2010). On the other hand, applying a current of a higher frequency, such as 140 Hz, shows excitatory effects (Moliadze et al. 2010). Testing of the tPCS protocol shows an increase in cortical excitability. However, according to Jaberzadeh et al. (2014), only pulses delivered in 500- and 250-millisecond ranges increased cortical excitability, while 120 milliseconds does not. Whether ranges of ≤120 milliseconds lead to inhibitory effects is not yet known. Wide-spectrum tRNS (0.1–640 Hz) has been documented to increase the overall cortical excitability (Terney et al. 2008, Moliadze et al. 2014), as the high frequency modulates the behaviour of the sodium channels (Bromm 1969). While the excitatory effects of tRNS are well documented, the inhibitory effects of tRNS are yet to be fully determined. For example, applying full-spectrum tRNS over the primary motor cortex with an intensity of 1.0 mA leads to an increase in excitability comparable to the excitatory effects of anodal tDSC; however, using wide-spectrum tRNS under an intensity of 0.4 mA significantly suppressed motor-evoked potentials compared to baseline and sham stimulation (Moliadze et al. 2012). Moliadze et al. (2012) also showed the possibility of enhancing or reducing cortical excitability by applying different intensities (1.0 and 0.4 mA) of tRNS, whereas Campana et al. (2016) showed the opposite effects of low- (0.1–100 Hz) and high-frequency (100–640 Hz) tRNS on visual motion adaptation. When applied to the human middle temporal (MT) complex, high-frequency tRNS causes a significant decrease in motion after-effect duration, while low-frequency tRNS causes an increase in motion after-effect duration.

#### Comparison of protocols

Inukai et al. (2016) compared the effectiveness of the three above-mentioned protocols: tDCS, tACS, and tRNS. The results showed that all of the protocols provide an increase (with respect to the prestimulation baseline) in cortical excitability. However, only tRNS showed increased motor-evoked potentials at all the measured time points (immediately after stimulation, 5 minutes, 10 minutes, and 20 minutes) compared to the sham stimulation (placebo condition), tDCS, and tACS. The conclusion of the study by Inukai et al. (2016) describes tRNS as being a superior protocol to tDCS and tACS in changing cortical excitability. A similar conclusion may also be found in other studies. Vanneste et al. (2013) showed that tRNS is more effective in tinnitus relief than tDCS and tACS. Compared to tDCS, the application of tRNS resulted in a longer motor-evoked potential (Moliadze et al. 2014), and possibly due to the many different frequencies used, it is more powerful than tACS (Inukai et al. 2016).

The mechanisms behind the power of tRNS are not yet completely understood. Some speculate about stochastic resonance (Stacey and Durand 2000), where a weak signal is amplified by adding noise. Another possible explanation comes from Antal and Herrmann (2016), who assume that the power of tRNS lies in the reduction of the amount of endogenous noise used by synchronization of neural activity through the amplification of oscillatory activity.

According to the above-mentioned studies, tRNS is superior to other protocols and may be used for both excitation and inhibition of cortical tissue. For inhibition, an intensity of up to 0.4 mA with a frequency ranging between 101 and 640 Hz is used, whereas for excitation of brain areas, an intensity of 1.0 mA with a frequency ranging between 0.1 and 100 Hz is used.

The second possibility is to utilize the tACS protocol within a range of specific frequencies, such as gamma or P300, which were previously considered as NCCs. However, recent studies falsified gamma activity (Jiang et al. 2006, Kanai et al. 2006, Hsieh et al. 2011) and P300 (Pitts et al. 2012, Silverstein et al. 2015) as NCCs, claiming they are actually markers of attention and novelty (Koch et al. 2016). tACS within these specific ranges may be used to add additional supportive evidence that gamma belongs to the mechanism of attention and not consciousness. Therefore, tRNS represents a logical choice as the best possible protocol due to its randomized ranges of neuromodulation frequencies for the causal alterations of excitation and inhibition.

Since we have the equipment for precise stimulation as well as the correct stimulation protocol, the next logical step is to consider where in the brain we should begin to uncover the overlapping functionally distinct neural processes of conscious experience. The simplest way to do this is to establish a set of basic testable hypotheses based on the current state of knowledge obtained from correlational and causal experiments. Such hypotheses will rest on specific brain regions, their functions, and connections that can be tested in a series of experiments. The following sections will provide examples of these hypotheses and experiments.

This will naturally ignite criticism of corticocentrism (see Solms 2021) and the localizationist framework (see Pessoa 2022). Corticocentrism is the belief that the cortex, or the outer layer of the brain, is the most important and dominant structure in the brain. However, the brain is a highly complex organ, and other structures, such as the subcortex, also play important and non-negligible roles in brain function. The localizationist framework in neuroscience is a theoretical perspective that suggests that specific cognitive functions, such as language, memory, or consciousness, are localized to specific regions or “modules” that are specialized for particular functions of the brain. Criticism of this framework is raised from the position of a distributed view of brain function, which suggests that cognitive processes are the result of the coordinated activity of many different brain regions working together.

These criticisms are legitimate, and there is no way to avoid them. However, if we consider what has been said earlier, then the use of GTEN can be partially shielded from them. GTEN can influence (to some extent) the deeper regions of the brain, and it is also able to influence several regions at the same time. In this way, it is possible to target specific brain regions as well as large-scale brain networks.

## Hypothesis of three distinct mechanisms of consciousness

**Figure 2.**
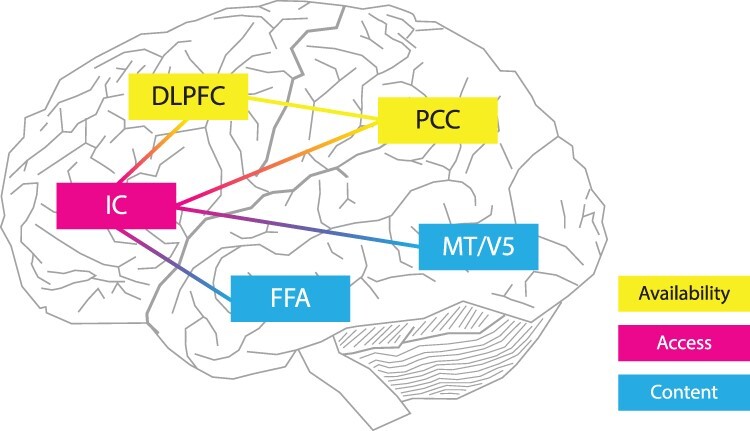
Functionally distinct neural mechanisms of consciousness

Let’s consider the following hypothesis: conscious experience as we know it is the effect of the causal chain of neural causes that are associated with functionally distinct neural mechanisms. To simplify the causal framework, let’s consider only three neural mechanisms: the mechanism of content, mechanism of access, and mechanism of availability, which contribute to the emergence of a united conscious experience (Fig. 2).

Three functionally distinct neural mechanisms are each associated with specific neural regions. The first mechanism is responsible for “specific contents” of conscious experience. This mechanism is constituted by specific sensory regions located in posterior brain areas (Mehta and Mashour 2013, Koch et al. 2016), each responsible for specific content such as faces (Tong et al. 1998, O’Craven and Kanwisher 2000, Barton and Cherkasova 2003, Rangarajan et al. 2014), objects (Malach et al. 1995, Grill-Spector et al. 2001, Wyart and Tallon-Baudry 2009, Emberson et al. 2017), motion (Zeki et al. 1993, Schenk and Zihl 1997, Kourtzi and Kanwisher 2000, Rauschecker et al. 2011), colour(s) (Zeki 1990, Barton 2011), and so on. The second mechanism is responsible for the “conscious access” or the so-called entry mechanism through which content gains access to conscious experience. This mechanism is located within the insular cortex (IC), considered the “core” system for sensory processing (Dosenbach et al. 2007), and is composed of several overlapping functions, such as perceptual recognition (Ploran et al. 2007), detection of salient contents (Yang et al. 2007, Menon 2015, Tamietto et al. 2015), and resolution of perceptual conflicts (Weilnhammer et al. 2021). The third mechanism is responsible for the “global availability” of conscious contents and also represents the ongoing stream of conscious experience into which specific contents enter. It sustains contents conscious for necessary periods of time and performs the function of the availability for use of conscious information by other systems, such as reasoning or action (Block 2005) or until a previous mechanism selects new and more salient content. This mechanism may be considered as general consciousness (Mehta and Mashour 2013) or the so-called full NCC (Koch et al. 2016) and is associated with the activity of post-sensory areas of the brain, namely the PPC and the dorsolateral prefrontal cortex (DLPFC), which together form the frontoparietal network (Dehaene 2014, Scolari et al. 2015, Marek and Dosenbach 2018).

### Evidence for the three distinct mechanisms of consciousness

As was stated previously, the causal explanation brings several obligations. The first one is that there should be at least some basic knowledge of which mechanisms produce specific behaviour or outputs within the complex system.

“Sensory regions” are the most probable for “mechanisms” of “contents”. For example, fusiform face area (FFA) is considered the sensory area that represents faces. Lesions of the FFA cause the so-called prosopagnosia, defined as a loss of the ability to see and recognize faces (Barton and Cherkasova 2003). The FFA function has also been confirmed by fMRI, either during active face stimulation (Tong et al. 1998) or during their imagination (O’Craven and Kanwisher 2000). Further evidence of the association of FFA with faces may be found in studies using electric stimulation. If the FFA is stimulated in this way, then conscious perception of faces is disrupted (Rangarajan et al. 2014).

Another example of specific content may be provided by the medial temporal cortex (MT/V5). MT/V5 is considered the sensory area responsible for motion. Lesions of the visual region of MT/V5 lead to the loss of this specific content (Schenk and Zihl 1997). MT/V5 is also active during the experience of illusory motion (Zeki et al. 1993), and the induction of TMS (Kourtzi and Kanwisher 2000) leads to the induction of the experience of motion (Rauschecker et al. 2011). Bilateral electric stimulation of MT/V5 leads to a significant decrease or increase in the duration of motion after-effect phenomena based on a selected frequency (Campana et al. 2016). Magnetic stimulation may either disrupt the processing of moving stimuli or even abolish it from conscious perception (de Graaf et al. 2011a, 2011b, Jacobs et al. 2012a, 2012b).

The lateral occipital complex (LOC) is defined as the low-level visual processing brain area that plays a role in the processing of shape and object stimuli (Grill-Spector et al. 2001). One of the first studies (Malach et al. 1995) reported that LOC responds strongly to everyday objects rather than visual textures without a clear interpretation of shape. Later studies further proved its role in shape processing, describing that LOC is highly selective for object shapes rather than texture or colour (Emberson et al. 2017). Also, gamma band activity (Wyart and Tallon-Baudry 2009) and activity between 200 and 300 milliseconds (Liu et al. 2012) were reported to influence seen or not seen stimuli in LOC.

Usually, damage to the specific sensory region abolishes the specific content from conscious experience such as the inability to experience colours (Zeki 1990), faces (Barton and Cherkasova 2003), or motion (Schenk and Zihl 1997), while the rest of the conscious experience is largely intact. This suggests that specific sensory areas may be considered as a minimal neural basis of the content of experience (Block 2005) related to aspects of phenomenal consciousness, such as the experience of red or pain. However, their activity or hyperactivity alone may not be sufficient for content to enter the conscious experience (Boly et al. 2004), and therefore, another mechanism is needed.

The “insular areas” have not been traditionally considered in discussions of “conscious access”, but some of their functions should definitely be implemented in the current discussions. For example, the IC plays an important role in the detection of salient phenomena from the external environment (Seeley et al. 2007, Corbetta et al. 2008) and is responsible for switching between the major brain networks, the default mode network, and the frontoparietal network (Menon 2015), each accompanied by different conscious contents. While the function of the frontoparietal network (Scolari et al. 2015) is associated with exteroceptive information processing, the default mode network is associated with interoceptive information processing (Raichle 2015) and various kinds of internal cognition, such as mind-wandering (Mason et al. 2007, Callard et al. 2013), mental time travel (Østby et al. 2012), and internal train of thought (Smallwood et al. 2012). IC is structurally connected with visual brain areas (Jakab et al. 2012) and von Economo neurons (Nimchinsky et al. 1999), which mediate rapid communication between distant brain regions. IC also receives inputs from other sensory systems and is also associated with attentional functions and working memory when a salient or deviant event is detected among a number of standard stimuli (Menon 2015). Meta-analyses have confirmed that the IC is activated across a number of different cognitive paradigms (Swick et al. 2011), and its activations during different cognitive tasks lead to the conclusion of its function as the possible “core” system for sensory processing (Dosenbach et al. 2007) with functions, such as perceptual recognition (Ploran et al. 2007) and generation of the perceptual choice (Thielscher and Pessoa 2007). The current question is how IC is related to awareness (Craig and Craig 2009) and conscious experience (Salomon et al. 2016). According to Vidal et al. (2015), IC not only contributes to the results of visual paradigms but also detects and resolves perceptual conflicts (Weilnhammer et al. 2021) and is thought to mediate access of visual information to conscious experience (Salomon et al. 2016), where more salient stimuli, such as fearful bodies, get preferential access to visual awareness (Tamietto et al. 2015).

The “third” and final mechanism is responsible for general consciousness and global availability. It is the ongoing stream of conscious experience that sustains various salient contents conscious. The most probable place to find this mechanism is in post-sensory brain areas, formed into the so-called frontoparietal network (Dehaene 2014, Scolari et al. 2015) consisting of the PPC and the DLPFC.

PPC functioning is connected to the seminal research of neglect (Marshall and Halligan 1988). Research shows that patients with lesions of the parietal cortex begin to neglect one side of their exteroceptive fields. Presented stimuli and mental content (e.g. a burning house) cannot reach consciousness; however, they still affect the patient’s behaviour. This recurrent behaviour of the participants in terms of their deficit (lesion) may be interpreted as a disruption of the mechanism responsible for “sustaining” content in the stream of general consciousness. Neglect may be artificially created by magnetic stimulation over the parietal cortex in healthy participants (Pascual-Leone et al. 1994a). The role of the parietal cortex in visual awareness has been demonstrated by several studies, using paradigms such as change blindness (Beck et al. 2006, Tseng et al. 2010), attentional blink (Kihara et al. 2007), and others (Zaretskaya et al. 2013). Further research fully congruent with these findings comes from stimulation studies. Application of TMS to the parietal areas of the brain may prolong (Zaretskaya et al. 2010) or vice versa shorten (Pearson et al. 2007, Carmel et al. 2010) the time for which individual stimuli are dominant. It may affect the rate of perceptual switching in bistable vision paradigms (Carmel et al. 2010, Kanai et al. 2011) and affect the maintenance of conscious percept, such as fading from conscious experience (Kanai et al. 2008). An inhibitory magnetic stimulation protocol increases the duration of target disappearance under conditions of a motion-induced blindness paradigm (Nuruki et al. 2013). Electric stimulation of the visual parietal cortex may inhibit the capacity to detect, localize, and focus on static visual targets (Schweid et al. 2008).

The role of the frontal regions and DLPFC in consciousness is still unclear even though their activity is regularly reported (Doesburg et al. 2009, Dehaene 2014). It has not been clarified yet whether DLPFC is necessary for the development of conscious experience or whether its activity is related to mere reporting or accompanying metacognition, i.e. the awareness of one’s own conscious mental states (Frässle et al. 2014). Studies have shown that even large bilateral lesions of the DLPFC have a negligible effect on conscious experience (Brickner 1952, Mataró et al. 2001, Kozuch 2014), and neither electric nor magnetic stimulation of frontal regions causes any substantial change in conscious content (Pascual‐Leone et al. 1991). On the other hand, according to other reports, lesions of the frontal cortex may result in visual neglect (Husain and Kennard 1996) analogous to lesions of the parietal areas. Congruently, the transcranial stimulation of DLPFC reduces the chance that the participant will report seeing the stimulus (Rounis et al. 2010) and detect the visual change (Turatto et al. 2004). Studies have also shown that synchronization between frontal and parietal brain areas is critical for the orientation of attention (Daitch et al. 2013) and maintenance of working memory (Salazar et al. 2012) and, for some (Prinz 2012), the necessary building blocks of conscious experience. Stimulation of DLPFC was also reported as being influential in the proportion of consciously perceived target stimuli under the attentional blink paradigm (London and Slagter 2015), and some even consider DLPFC as the neural marker of conscious access (Sterzer and Kleinschmidt 2007, Panagiotaropoulos et al. 2012). Stimulation of DLPFC is also promising as a rehabilitation procedure in disorders of consciousness (Angelakis et al. 2014) as well as in tinnitus-related distress relief (Vanneste and De Ridder 2011, Frank et al. 2012).

Considering the conflicting conclusions, it is possible that DLPFC has some but not a central role in sustaining content conscious, and the “heavy lifting” is performed mainly by PPC.

As mentioned above, the hypothesis of three distinct and functionally overlapping mechanisms was introduced with brain areas that are the most suitable candidates for them. However, this hypothesis cannot be proved (or disproved) using the correlational methodology and cannot be proved at one time. Proving and disproving this hypothesis, the same as the possible localization of each mechanism (and their further decomposition on lower levels), require it to be separated into two different (but related) series of research experiments: inhibitory experiments and excitatory experiments.

### Limitations

We acknowledge that our proposed mechanisms in the above-mentioned sections are not a complete mechanistic explanation of consciousness but rather a hypothesis about the brain regions where certain functions related to conscious content, access, and availability may be performed. Before attempting to explain the underlying mechanisms at lower levels, it is necessary to establish the causal structures among these higher-level functions of consciousness. A fuller mechanistic explanation would require not only an understanding of these higher-level functions and the underlying mechanisms at lower levels but also an explanation of the causal interactions between these lower levels. Unfortunately, such an explanation is currently beyond the scope of both our current study and the current state of the art and available technology. However, we hope that our hypothesis will provide a useful starting point for further research on the mechanisms of consciousness.

### Experimental approach to the causal models of consciousness

To remediate the principal limitations of correlational methodology in the research of consciousness, the proposed causal approach based on neurostimulation techniques should be utilized and divided into a series of inhibitory and excitatory experiments.

“Inhibitory experiments” should focus on the disentangling of functionally distinct mechanisms of conscious experience. The series of inhibitory experiments should gradually target the above-reviewed sensory regions, insular regions, and finally post-sensory regions to determine their different roles in conscious experience. First, inhibitory experiments should focus on the inhibition of sensory brain areas to determine whether their roles are solely the creation of phenomenal representation or they go beyond this function and participate also in subsequent functions. If the inhibition only disrupts the phenomenological structure of the content without affecting any other conscious functions, such as access or availability, it is possible to claim that sensory regions are involved solely in the creation of the phenomenological aspects of conscious content. Second, inhibitory experiments should also focus on the inhibition of insular regions to determine their role as the access mechanism in conscious experience, their involvement, and functions regarding choosing specific and the most salient content (provided by sensory regions). If the inhibition of the IC is associated with impaired function of access of specific content to conscious experience without disruption of its phenomenal structure, it is possible to claim that insular regions are involved solely in the access mechanisms. Finally, inhibitory experiments should focus on the inhibition of frontal and parietal brain areas to determine what role their coactivity plays in consciousness. The inhibition of these areas should shorten the dominance or overall availability of specific content.

On the other hand, “excitatory experiments” should focus on finding the minimal sets of neural conditions necessary for perceptual content to become conscious (Crick and Koch 1990, 2003). However, in this case, the aim should not be to describe the difference in conditions between conscious content and unconsciousness content but to directly describe the causal neural conditions responsible for the specific stimulus entering the conscious experience. The series of excitatory experiments should gradually target sensory regions, insular regions, and finally frontal and parietal areas of the brain to determine the minimum necessary interconnection of neural mechanisms that is sufficient to make a specific content conscious.

Excitatory experiments should start with sensory brain areas. While disturbing the specific sensory region disturbs a content’s phenomenal features, it does not mean that the activity of the sensory region alone is sufficient for the content’s entry into conscious experience. Of course, excitatory experiments should determine whether the excitation of the specific sensory brain area is sufficient for a content to become conscious or dominant among other competing contents, but this does not seem likely. For example, a recent study of tinnitus has demonstrated that hyperactivity of the sensory area alone is not sufficient for its conscious perception, and synchronized co-activation of frontal and parietal brain regions is necessary for its conscious perception (Boly et al. 2004).

The second step should be the excitatory co-activation of the sensory brain area and insular regions to determine whether their artificially induced co-activation is sufficient for specific content to enter conscious experience. In this case, it is possible to assume that such co-excitation would be sufficient for content to enter conscious experience. However, under such conditions content may appear to the individual as a glimpse that would not be part of the conscious experience for long. It is possible that for the long-term availability of the specific content within conscious experience, additional coactivity is needed.

Therefore, it is necessary to implement the third part of excitatory experiments. These should focus on the co-excitation of the sensory brain area, insular regions, and post-sensory brain areas to determine whether their artificially induced co-activation is sufficient for content to not only become part of the conscious experience but also increase its overall availability. The overall availability of specific content should be determined by comparison of time series during which the stimulus was consciously perceived between two different excitation experiments, i.e. experiments focusing on the co-excitation of sensory and insular regions and experiments focusing on the co-excitation of sensory, insular, and post-sensory regions.

Together, these series of inhibitory and excitatory experiments should not only provide an understanding of the minimal sets of neural events necessary for the content to become conscious but also describe the different functions performed by various mechanisms necessary for a united conscious experience.

## Conclusion

Conscious experience represents one of the most elusive problems of current empirical science. This elusiveness is primarily a consequence of correlational methodology, which is incapable of localizing and subsequently disentangling the various overlapping and functionally different mechanisms of conscious experience. The hypothesis of three distinct neural mechanisms of conscious experience cannot be proved or disproved by observational research based on correlational methodology but only by the methodology of causal alterations via analytical methods of excitation and inhibition. Electric brain stimulation with the use of GTEN technology and its excitatory and inhibitory effects on the underlying brain structures is one possible way of testing the above-mentioned hypotheses. If successful, gradual excitatory and inhibitory experiments will become the stepping stones for the localization of mechanisms of consciousness and therefore the basis for any subsequent research of consciousness and conscious mental states.
